# Supplementary material for: Immune responses and disease biomarker long-term changes following COVID-19 mRNA vaccination in a cohort of rheumatic disease patients
Source: Front Immunol. 2023 Jul 31;14:1224702. doi: 10.3389/fimmu.2023.1224702 (PMC10424846; doi:10.3389/fimmu.2023.1224702)
Supplement: Supplementary file 1 [file Table_1.docx]

**Supplementary Table 1.** Characteristics of patients with rheumatic diseases.

|  | **Number of Patients (N= 28)** | **Percentage (%)** |
| --- | --- | --- |
| **Gender** |  |  |
| Female | 20 | 71.42 |
| Male | 8 | 28.57 |
| **Age (years), mean (SD)** | 53 (+/-12) |  |
| 20-29 | 2 | 7.14 |
| 30-39 | 2 | 7.14 |
| 40-49 | 7 | 25 |
| 50-59 | 8 | 28.57 |
| 60-69 | 7 | 25 |
| 70-79 | 1 | 3.57 |
| 80-89 | 1 | 3.57 |
| **Rheumatic disease** |  |  |
| Systemic lupus erythematosus (SLE) | 12 | 42.85 |
| Psoriatic Arthritis (PsA) | 10 | 35.71 |
| Sjogren's syndrome | 4 | 14.28 |
| Idiopathic Inflammatory Myopathy | 1 | 3.57 |
| Ankylosing Spondylitis | 2 | 7.14 |
| **Disease Activity (Measured at Visit 1)** |  |  |
| mLLDAS (SLE)* (n=12) | 6 | 50 |
| Active PsA^†^ (n=10) | 4 | 40 |
| **Visit** |  |  |
| 3 Visit | 15 | 53.57 |
| 4 Visit | 13 | 46.42 |
| **Vaccine** |  |  |
| PFIZER | 14 | 50 |
| MODERNA | 14 | 50 |
| **Treatments** |  |  |
| Prednisone | 10 | 35.71 |
| Methotrexate | 6 | 21.42 |
| Hydroxychloroquine | 11 | 39.28 |
| Mycophenolate | 3 | 10.71 |
| Azathioprine | 5 | 17.85 |
| Leflunomide | 3 | 10.71 |
| Tofacitinib | 1 | 3.57 |
| Adalimumab | 2 | 7.14 |
| Etanercept | 1 | 3.57 |
| Rituximab | 2 | 7.14 |
| Secukinumab | 4 | 14.28 |
| Belimumab | 1 | 3.57 |
| Ixekizumab | 1 | 3.57 |
| No DMARDs or biologics | 3 | 10.71 |

Note: One patient was diagnosed with both SLE and PsA.

*Modified lupus low disease activity status (mLLDAS) definition: SLEDAI-2K score of ≤4, with no activity in major organ systems, with no new features of disease activity compared with the previous assessment, prednisone of ≤7.5 mg/day and/or immunosuppressive drugs at maintenance dose at the visit date.

^†^Based on tender and swollen joint counts, new extra-articular involvement, and an increase in treatment.

**Supplementary Table 2.** Primer sequences used for PCR.

| Name | Primers for PCR (5’-3’) |
| --- | --- |
| *MX1* | Forward GTTTCCGAAGTGGACATCGCA |
|  | Reverse CTGCACAGGTTGTTCTCAGC |
| *IFIT1* | Forward AGAAGCAGGCAATCACAGAAAA |
|  | Reverse CTGAAACCGACCATAGTGGAAAT |
| *IFI44* | Forward TGGTACATGTGGCTTTGCTC |
|  | Reverse CCACCGAGATGTCAGAAAGAG |

**Supplementary Table 3:** Antibodies used in Flow Cytometry Analysis

| **Antibody target** | **Fluorochrome conjugate** | **Clone** | **Supplier** | | **Catalog #** |
| --- | --- | --- | --- | --- | --- |
| viability | FVS 620 |  | BD Biosciences | | 564996 |
| viability | BV510 |  | Tonbo Biosciences | | 13-0870-T100 |
| PD1 | BV711 | EH12.2H7 | Biolegend | | 329928 |
| IgM | percp-cy5.5 | MHM-88 | Biolegend | | 314512 |
| IgD | FITC | IA6-2 | Biolegend | | 348206 |
| HLA-DR | percp-cy5.5 | AC122 | Miltenyi Biotec | | 130095291 |
| CXCR5 | Alexa Fluor® 647 | J252D4 | Biolegend | | 356905 |
| CD8 | BV510 | RPA-T8 | Biolegend | | 301048 |
| CD56 | FITC | NCAM16.2 | BD Biosciences | 340723 | |
| CD45RA | APC-Cy7 | HI100 | Tonbo | | 25-0458-T100 |
| CD4 | AF700 | RPA-T4 | Biolegend | | 300526 |
| CD38 | Pac-blue | HIT2 | Biolegend | | 303525 |
| CD38 | BV421 | HIT2 | Biolegend | | 303525 |
| CD3 | BV650 (Biolegend) | OKT3 | Biolegend | | 317324 |
| CD3 | SB600 | OKT3 | Invitrogen | | 63-0037-42 |
| CD3 | BV650 | OKT3 | Biolegend | | 317324 |
| CD27 | PE-Cy7 | M-T271 | Biolegend | | 356412 |
| CD25 | BV785 | BC96 | Biolegend | | 302638 |
| CD24 | PE | ML5 | Biolegend | | 311106 |
| CD21 | APC | B-ly4 | BD Biosciences | | 561357 |
| CD20 | PE | 2H7 | Biolegend | | 302306 |
| CD19 | BV650 | HIB19 | Biolegend | | 302238 |
| CD14 | BV785 | M5E2 | Biolegend | | 301840 |
| CD138 | APC-Cy7 | MI15 | Biolegend | | 356528 |
| CD127 | BV605 | A019D5 | Biolegend | | 351334 |
| CD11c | RedFluor 710 | 3.9 | Tonbo Biosciences | | 80-0116 |
| CCR7 | PE-Cy7 | G043H7 | Biolegend | | 353226 |
|  |  |  |  | |  |

**Supplementary Table 4:** SLEDAI scores of each SLE patient.

|  | **SLEDAI scores** | | | |
| --- | --- | --- | --- | --- |
|  | **Visit 1** | **Visit 2** | **Visit 3** | **Visit 4** |
| **SLE-01** | 8 | 0 | 4 | -- |
| **SLE-02** | 2 | 5 | 3 | -- |
| **SLE-03** | 6 | 10 | 6 | 2 |
| **SLE-04** | 20 | 12 | 14 | 10 |
| **SLE-05** | 0 | 0 | 0 | -- |
| **SLE-06** | 0 | 0 | 4* | -- |
| **SLE-07** | 0 | 0 | 0 | 0 |
| **SLE-08** | 2 | 2 | 2 | 2 |
| **SLE-09** | 0 | -- | -- | 0 |
| **SLE-10** | 11 | 4 | -- | 4 |
| **SLE-11** | 0 | -- | 6* | -- |
| **SLE-12** | -- | 6 | -- | 6 |

*Representing arthritis.
