# Supplementary figures and images for: Immune responses and disease biomarker long-term changes following COVID-19 mRNA vaccination in a cohort of rheumatic disease patients
Source: Front Immunol. 2023 Jul 31;14:1224702. doi: 10.3389/fimmu.2023.1224702 (PMC10424846; doi:10.3389/fimmu.2023.1224702)

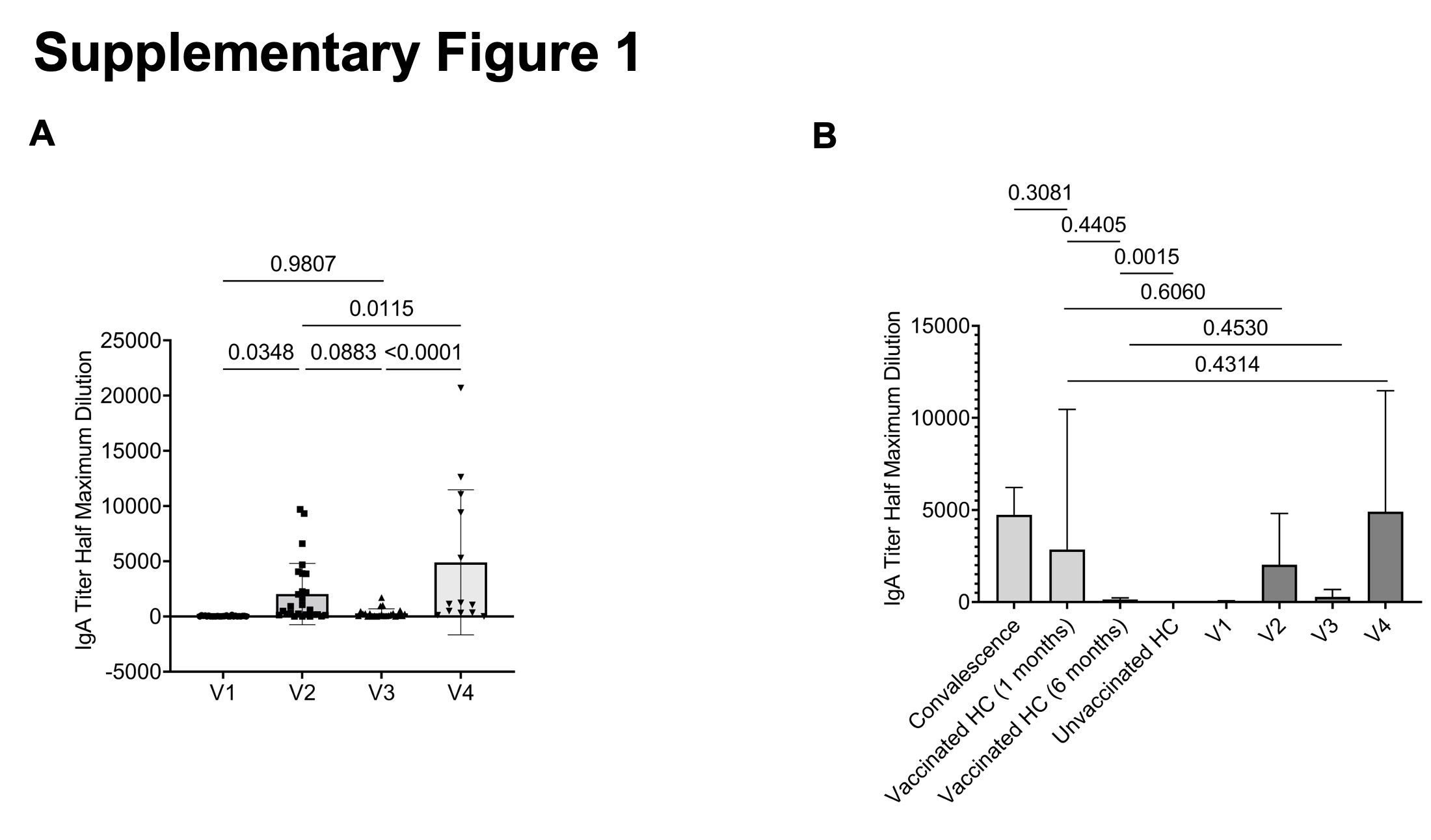

Supplement: Supplementary Figure 1 — Statistical analyses of antibody titer data between visits. (A), Comparisons of IgA titers half maximum dilution in rheumatic disease patients’ plasma samples between different visits. (B), Comparisons of IgA titers half maximum dilution among indicated groups. P values were derived from paired t-tests (A) and unpaired t-tests (B). [file Image_1.jpeg]

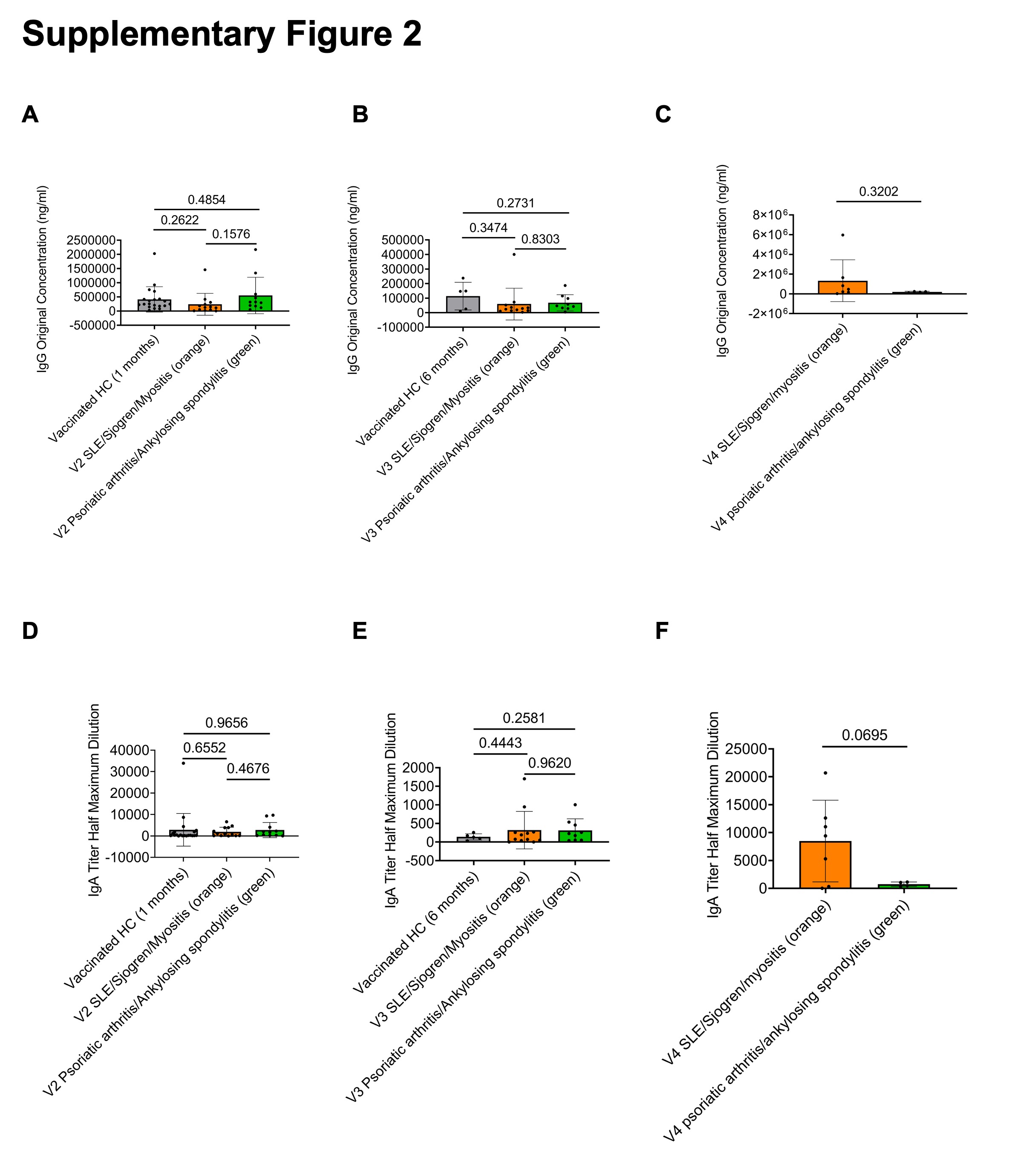

Supplement: Supplementary Figure 2 — Statistical analyses of antibody titer between disease groups at different visits. (A-C), Comparisons of anti-SARS-CoV-2 S1 protein IgG concentration between HC (gray), SLE/Sjogren/Myostitis (orange), and Psoriatic arthritis/Ankylosing spondylitis (green) at V2 (A), V3 (B) and V4 (C). (D-F), Comparisons of anti-SARS-CoV-2 S1 protein IgA titers between HC (gray), SLE/Sjogren/Myostitis (orange), and Psoriatic arthritis/Ankylosing spondylitis (green) at V2 (D), V3 (E) and V4 (F). P values were calculated by unpaired parametric t-test. [file Image_2.jpeg]

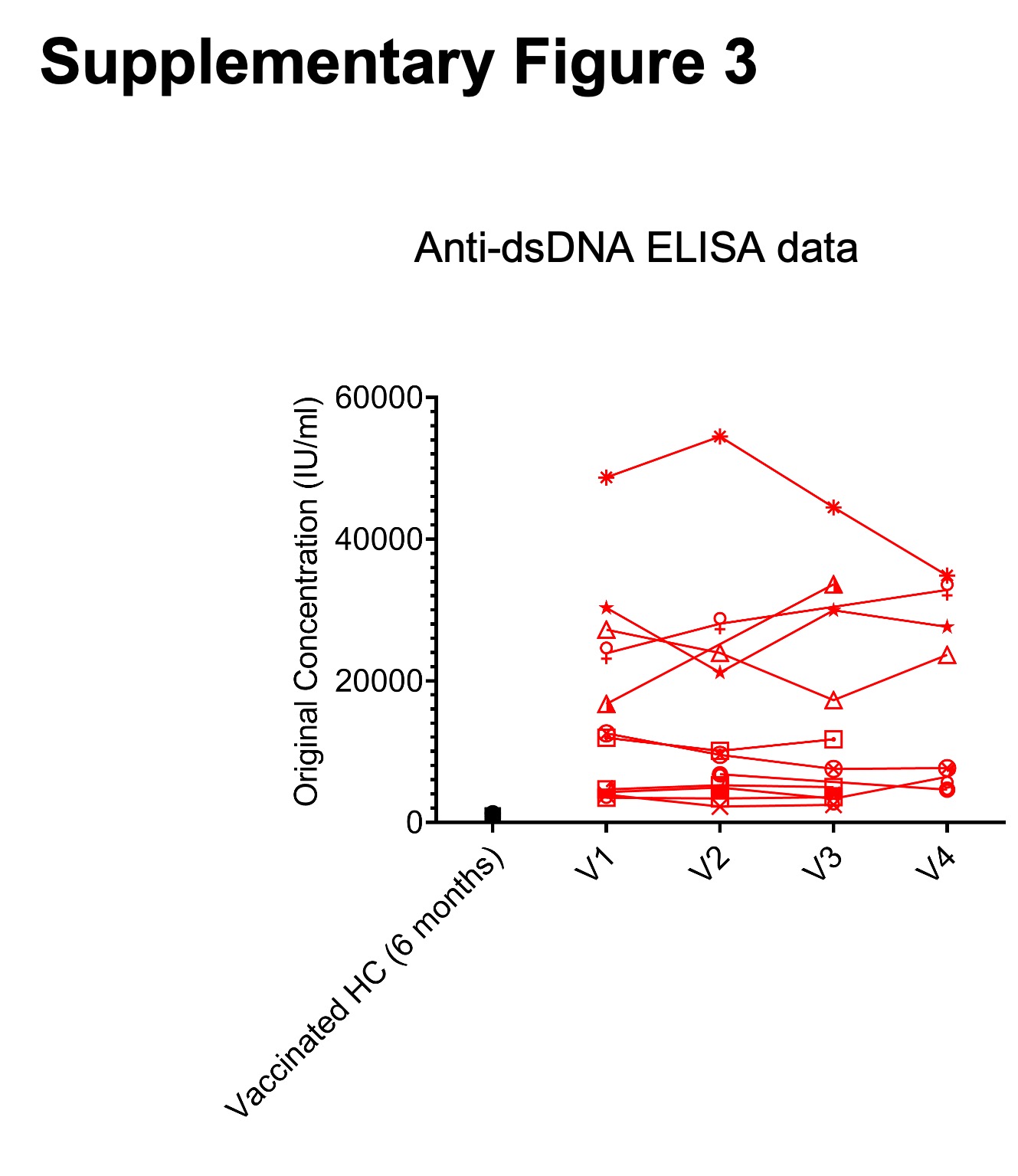

Supplement: Supplementary Figure 3 — Human plasma anti-double stranded deoxyribonucleic acid (anti-dsDNA) level. Concentration of anti-dsDNA in human plasma samples was obtained by ELISA. SLE patients (Red, n = 12). Healthy controls post-vaccinated six months (Black, n = 5). [file Image_3.jpeg]

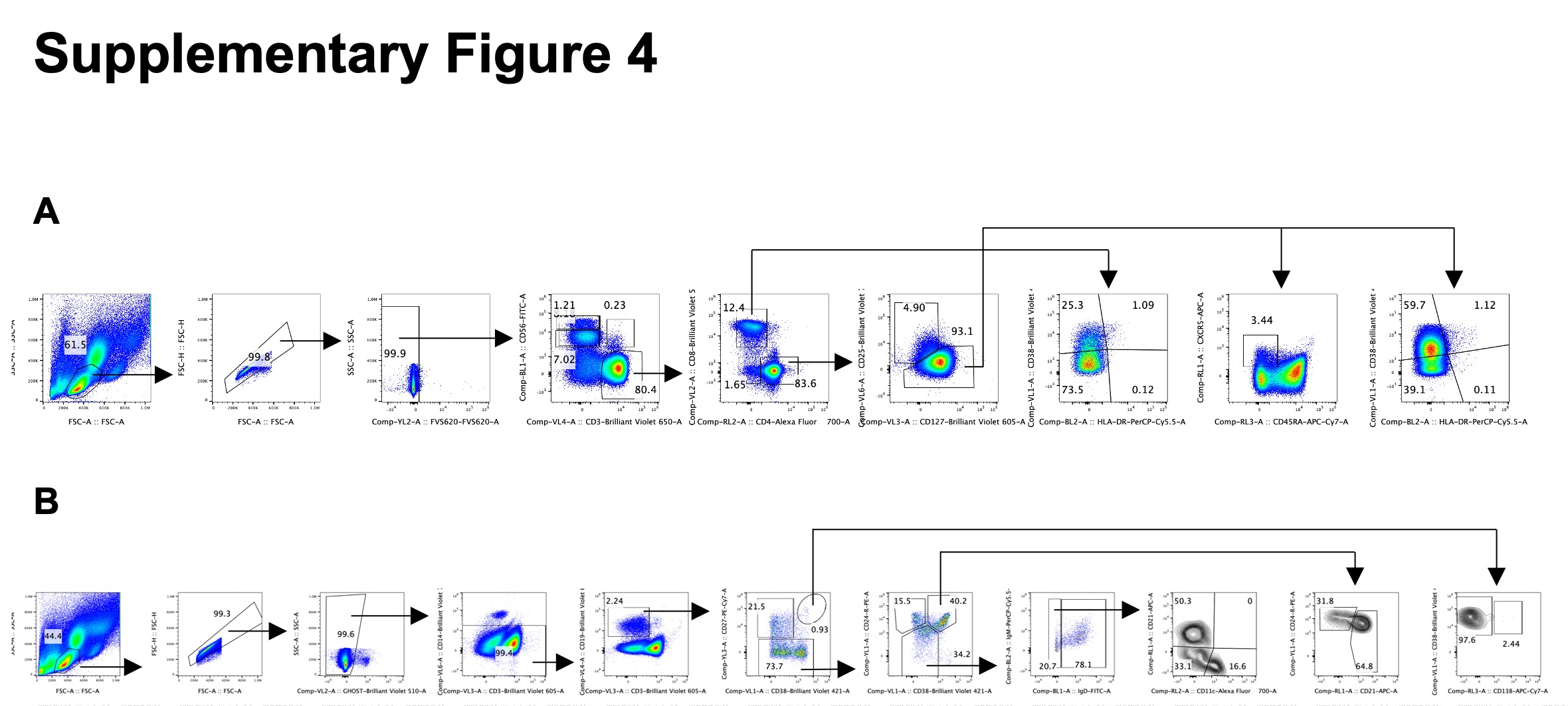

Supplement: Supplementary Figure 4 — Representative flow cytometry plots of the gating strategies. (A), Gating strategies for T cell subsets. (B), Gating strategies for B cell subsets. [file Image_4.jpeg]

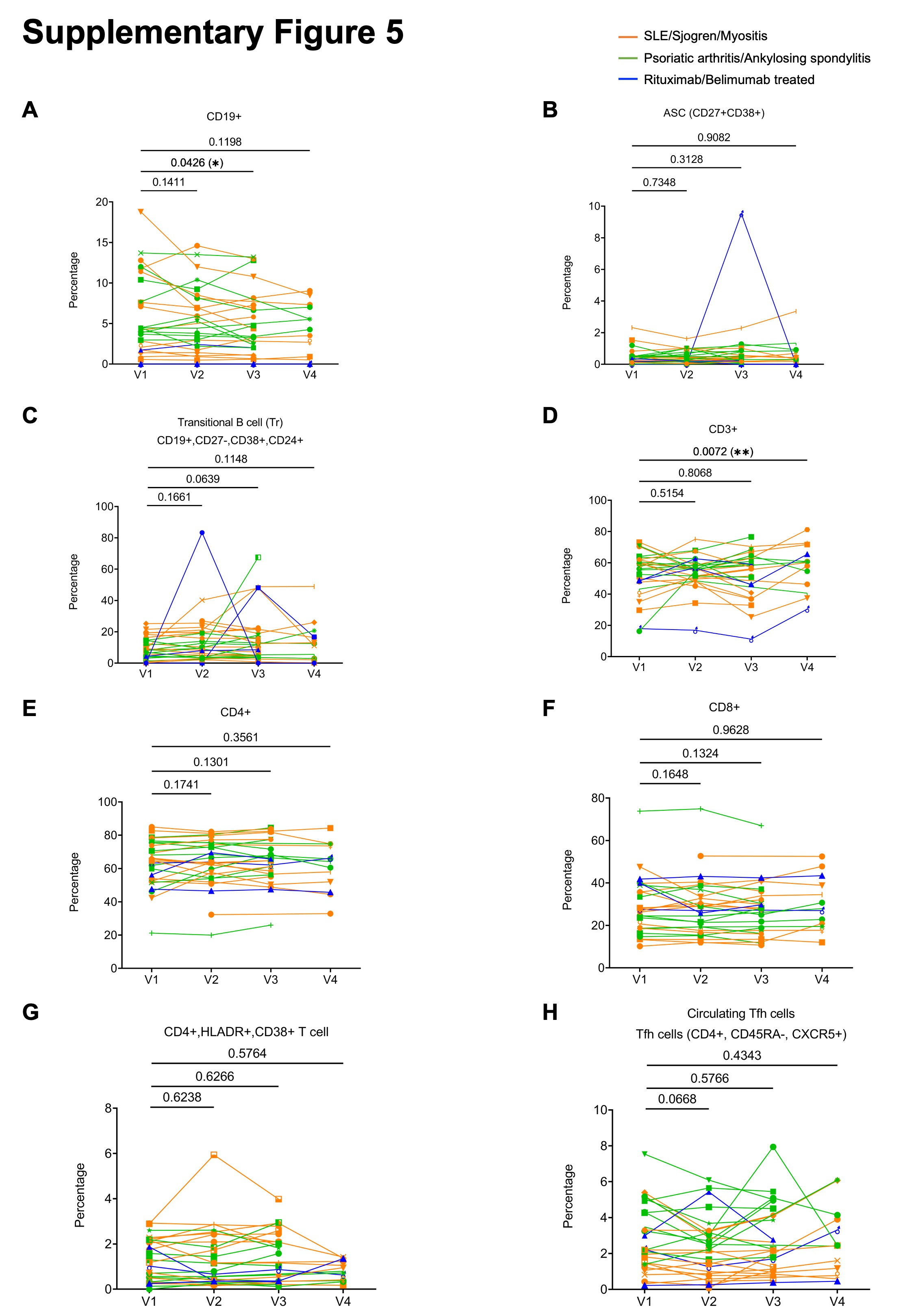

Supplement: Supplementary Figure 5 — Summaries of the immune cell subset frequencies in rheumatic disease patients’ PBMC samples following SARS-CoV-2 mRNA vaccination. (A, B) cells (CD19+). (B), Antibody secreting cells (ASC, CD19+CD27+CD38+). (C), Transitional B cells (CD19+CD27-CD38+CD24+). (D), T cells (CD3+). (E), CD4+ T cells (CD3+CD4+). (F), CD8+ T cells (CD3+CD8+). (G), TCR activated conventional CD4+ T cells (CD4+CD25–CD127+HLADR+CD38+). (H), Circulating follicular helper T cell (Tfh)-like cells (CD3+CD4+CD45RA–CXCR5+). P values were calculated by paired parametric t-test. Orange subgroup (n = 13). Green subgroup (n = 12). Blue subgroup (n = 3). [file Image_5.jpeg]
